# Risk-Based Fault Detection Using Bayesian Networks Based on Failure Mode and Effect Analysis

**DOI:** 10.3390/s24113511

**Published:** 2024-05-29

**Authors:** Bálint Levente Tarcsay, Ágnes Bárkányi, Sándor Németh, Tibor Chován, László Lovas, Attila Egedy

**Affiliations:** 1 Department of Process Engineering, University of Pannonia, 8200 Veszprém, Hungary; 2 Hungarian Gas Storage Ltd., 1138 Budapest, Hungary

**Keywords:** fault detection, dynamic risk assessment, Bayesian networks, FMEA, DPCA

## Abstract

In this article, the authors focus on the introduction of a hybrid method for risk-based fault detection (FD) using dynamic principal component analysis (DPCA) and failure method and effect analysis (FMEA) based Bayesian networks (BNs). The FD problem has garnered great interest in industrial application, yet methods for integrating process risk into the detection procedure are still scarce. It is, however, critical to assess the risk each possible process fault holds to differentiate between non-safety-critical and safety-critical abnormalities and thus minimize alarm rates. The proposed method utilizes a BN established through FMEA analysis of the supervised process and the results of dynamical principal component analysis to estimate a modified risk priority number (*RPN*) of different process states. The *RPN* is used parallel to the FD procedure, incorporating the results of both to differentiate between process abnormalities and highlight critical issues. The method is showcased using an industrial benchmark problem as well as the model of a reactor utilized in the emerging liquid organic hydrogen carrier (LOHC) technology.

## 1. Introduction

In this work, the authors introduce a hybrid framework of fault detection and risk assessment techniques which utilizes a modified risk priority number (*RPN*) to highlight safety-critical process abnormalities and minimize superfluous alarm rate. A combination of dynamic principal component analysis (DPCA) for fault detection (FD) and a failure mode and effect analysis (FMEA) based Bayesian network (BN) for risk assessment is proposed. The two methods work in parallel, utilizing each other’s results to pinpoint the presence of abnormalities and the simultaneous estimation of risk associated with the abnormalities through an *RPN* based on the results of both approaches. Only safety-critical process states are highlighted through alarm signals, while non-safety-critical process states where faults are present are only shown by warnings. The method thus mitigates alarm floods by eliminating alarms for faults which hold little risk to the process performance and safety going forward.

In the past, various techniques have been proposed for FD, and their performance has been continuously enhanced since to minimize the false alarm rate (FAR) and missed alarm rate (MAR) and allow for subsequent crisp fault detection using model- [1], data- [2] and qualitative knowledge-based [3] logic. While these techniques and, specifically, multivariate statistical process monitoring [4] methods among data-based techniques have adequate performance for system supervision and great popularity in the FD community, the problem with many FD methods as noted by multiple authors is the fact that they usually do not take the risk associated with each fault into account [5].

Among the multivariate statistical process monitoring (MSPM) techniques, the most popular, principal component analysis (PCA) based methods, utilize $T^{2}$ and *Q* statistics for FD and compare these metrics calculated from process data to predefined statistical thresholds to decide whether a sample can be categorized as normal or abnormal [6]. The performance of the FD method therefore is usually evaluated using only the FAR and MAR metrics. The issue with this approach is that since no risk is associated with the out-of-control process states in traditional MSPM FD, alarms will be raised regardless of whether the detected abnormality is just a simple nuisance that holds no process risk or if it is a state that could cause severe damage if left unchecked [7]. Therefore, many nuisance alarms are raised, which could lead to alarm floods in complex systems, especially when, due to fault propagation, other alarms are raised as well [8].

To circumvent this issue, methods have been developed that take the risk of each indicated fault into account during the FD process through dynamic risk assessment (DRA); however, the arsenal of techniques which utilize risk assessment in coordination with FD is still sparse [9]. Among the first instances of such methods was a technique which proposed a PCA model for the supervision of chemical processes and incorporated risk estimation using a quantitative risk assessment model. Using this technique, alarms were only raised when a fault was detected by the PCA metrics and the predicted risk exceeded a defined threshold [7]. Later on, self-organizing maps were utilized to address the FD problem of non-linear systems. Using a probabilistic approach, faults were categorized into several classes based on severity, and FD was performed while taking the risk into account as well [10]. Qualitative models have also become researched for the risk-based FD methodology, such as the use of the R-vine copula and the event tree methods for the supervision of non-linear and non-Gaussian processes [11].

The techniques for risk-based FD have become increasingly more researched and popular as the previous examples show but are still relatively scarce. While most methods propose risk estimation techniques which are in a manner related to traditional techniques of industrial risk assessment, such as hazard and operability study (HAZOP) [12], event trees (ETs) [13], fault trees (FTs) [14] or FMEA [15], these methods are not intrinsically integrated into the framework and performed rigorously [7].

For example, in the previously noted articles [5,7,10], the general definition of risk was formulated as a product of the probability of a fault occurring, which leads to an unwanted catastrophic event (*P*) and the severity score assigned to each fault consequence (*S*) as per Equation (1), from which a dynamic risk profile for the process was calculated [16]. This procedure and its basic logic are fairly similar to the calculation of *RPN* in FMEA:

(1) Risk(t)=P(t)·S

The probability of fault occurrence leading to catastrophic events was fitted as a cumulative normal distribution function in these cases, with the probability of catastrophe increasing as the process variables deviate from their expected values. Severity scores were calculated based on the type of process variable deviating, with each process variable having an assigned severity parameter and the *S* score being defined as a sum of the product of severity parameter and a function of the deviation of each variable from their normal value [5]. While the method proved applicable especially when combined with latent variable modeling techniques such as PCA [7] or self-organizing maps [10] where the latent variables were used for risk estimation, the approach has an issue in the case of real-world industrial application.

Since the probability of fault occurrence which could lead to catastrophic results was fitted as a cumulative normal distribution function, the probability of faults leading to catastrophic events would change uniformly regardless of which variables deviate. From a general process perspective, it is obvious that the probability of fault occurrence which could lead to catastrophic results is dependent not only on the magnitude of the process variable deviation from its normal state but also on the type of process variables which deviated [16].

For example, in industrial systems, for safety-critical process variables such as temperature or pressure, often, inherent safety protocols or fail-safes are in place which significantly lower the probability of catastrophic events occurring even if, due to some fault, a critical process variable shows abnormal behavior [17]. Therefore, the probability of safety-critical events occurring may not be properly characterized by simply observing the deviation of process variables from their normal states without taking the general construction of the system, presence of fail-safes, inherent safety, and possible fault propagation paths into account.

To overcome this issue, established risk assessment methods and models from the literature have been evaluated to propose hybrid techniques for more rigorous risk-based FD [18]. Based on previous trends, the most popular methods for quantitative risk assessment are probabilistic graphic models, such as dynamic event and fault trees, event sequence diagrams, Markov models [19], Monte Carlo simulation [20], BNs, Petri nets [21], etc., to estimate the risk of certain system states under both static and dynamic conditions [22].

In recent years, BNs have gained especially great popularity in the risk assessment community, with many applications aiming to extend their applicability and combining them with previously established methods such as FMEA or ETs [23]. The allure of these techniques is a more rigorous way to estimate the probability of process risks than the traditional FMEA or HAZOP techniques and addressing the entirety of the system (fail-safes and components included) and taking possible failure propagation paths into account [23].

In light of this, the key idea of this article is to extend the framework of risk-based FD using a method based on dynamic principal component analysis (DPCA) and BN-FMEA-based risk assessment, which can be used to give a more accurate estimate of process risk by taking fault propagation paths into account as well (fail-safes and inherent safety as well) when evaluating possible abnormal process states.

The authors utilize DPCA to characterize the observed process and produce indicators for the presence of process faults and risk events. After establishing the model under normal operating conditions, the presence of characteristic faults is observed, and statistic indicators such as the *Q* statistic are calculated for the different fault scenarios. Parallel to the FD procedure, a risk profile is observed for the system based on BN-FMEA. Severity scores are assigned based on the deviating principal components, detectability is evaluated using MAR metrics of the DPCA technique, and the probability of fault presence is evaluated using the BN. This approach results in a modified *RPN*, which is used to indicate whether a process state poses significant risk to process operations or not through alarm and warning signals. In the paper, the following definitions of alarm and warning signals are used:

- Alarms: Signals with intense visual and vocal prompts used to signal operators that a shutdown of the supervised system or other immediate and severe actions are necessary.
- Warnings: Signals with vocal and visual prompts which signal to operators that process functions are lost/process states changed due to process faults, but immediate shutdown is not necessary, as the disturbances are not critical from a safety perspective.

As can be seen, safety-critical system states are highlighted using alarm signals, while non-safety-critical process conditions are still recognized by warning signals. Going forward, the main contribution of our approach can be summarized in the following points.

- Development of a risk-based fault detection method which combines standardized expert knowledge (failure mode and effect analysis) with data-based techniques (Bayesian network) for risk assessment.
- Introduction of a modified *RPN* containing both FD and risk assessment consideration for the raising of alarms.

In the following, the mathematical formalization and background of the employed techniques are introduced in Section 2. The flowchart and general logic of the proposed algorithm are formalized is Section 3. Case studies for method evaluation are given in Section 4, including both a case study of an FD benchmark problem and a case study utilizing a dehydrogenation reactor of the liquid organic hydrogen carrier (LOHC) technology. The discussion and critical evaluation of the results are shown in Section 5.

## 2. The Proposed Risk-Based FD Method and Utilized Techniques

In this section, the basic techniques of DPCA, FMEA and BN are introduced and formalized; subsequently, the proposed technique utilizing the methods for risk-based FD is explained.

### 2.1. Principal Component Analysis (PCA) and Dynamic Principal Component Analysis (DPCA)

Consider a data set describing the behavior of a process, denoted by $X\in R^{n\times p}$ with *n* observations and *p* process variables. The columns of X are centered and scaled to have a mean of zero and unit variance. The centered and scaled X matrix shall be denoted as $\tilde{X}$. The sample covariance matrix $Z\in R^{p\times p}$ of $\tilde{X}$ may be calculated according to Equation (2):

(2) $$Z=\frac{1}{n-1}{\tilde{X}}^{T}\tilde{X}$$

The eigenvalue decomposition of the covariance matrix Z according to Equation (3) results in $P\in R^{p\times p}$, which is a matrix containing the eigenvectors of Z, while $\Lambda \in R^{p\times p}$ is a diagonal matrix containing the eigenvalues of Z.

(3) $$Z=P\Lambda P^{T}$$

After arranging the eigenvectors based on the value of their corresponding eigenvalues in descending order, we obtain the matrix $\tilde{P}$. The optimal number of PCs to be retained $\left(a\right)$ can be calculated according to Equation (4), where θ is the cumulative value of the eigenvalues to be retained, provided that $\sum_{i=1}^{p}{\tilde{v}}_{i}\geq \theta$ holds true:

(4) $$a=\arg\min_{i}{\left(\sum_{i=1}^{q}{\tilde{\lambda }}_{i}-\theta \right)}^{2}$$

The PCA transformation is then realized in the form of Equation (5) with $T\in R^{n\times a}$:

(5) $$T=\tilde{X}{\tilde{P}}_{a}$$

The PCA data decomposition model thus takes the form shown in Equation (6), where the matrix $E\in R^{n\times p}$ is the prediction error:

(6) $$\tilde{X}=T{\tilde{P}}_{a}^{T}+E$$

After dimensionality reduction, control statistics such as the Hotellings $T^{2}$ statistic or the *Q* statistic can be used for system supervision [24]. The $T^{2}$ statistic measures a sample’s variance within the expectance of the model and detects samples with great deviation as outliers. On the other hand, the *Q* statistic, also known as Squared Prediction Error (SPE), provides a measure for the prediction error of the PCA model for a given data point and classifies data points which do not follow the model as outliers regardless of their variance [25]. The $T^{2}$ statistic for a given *i*-th sample can be calculated according to Equation (7) using the calculated principal components:

(7) $$T_{i}^{2}=T_{i}\Lambda^{-1}T_{i}$$

Assuming that the PCs are normally distributed, an upper control limit can be established, which can be used to filter abnormal outlier points. In the case of the $T^{2}$ statistic for a given confidence level α [26], the control limit $T_{\alpha }^{2}$ may be calculated according to Equation (8), where *F* is the *F*-distribution [27]:

(8) $$T_{\alpha }^{2}=\frac{a(n+1)(n-1)}{n^{2}-na}F\left(\alpha ,a,\left(n-a\right)\right)$$

The *Q* statistic for an *i*-th data point can be calculated according to Equation (9), where $I\in R^{p\times p}$ is a unit matrix with appropriate dimensions [26]:

(9) $$Q_{i}={\left({\tilde{X}}_{i}-T{\tilde{P}}_{a}^{T}\right)}^{T}\left({\tilde{X}}_{i}-T{\tilde{P}}_{a}^{T}\right)={\tilde{X}}_{i}^{T}\left(I-{\tilde{P}}_{a}{\tilde{P}}_{a}^{T}\right){\tilde{X}}_{i}=E_{i}^{T}E_{i}$$

A control metric for the *Q* statistic has been proposed by Jackson and Mudholkar [26]. For a given confidence level, α the control limit $Q_{\alpha }$ is calculated according to Equation (10):

(10) $$Q_{\alpha }=\theta_{1}{\left[1+\frac{d_{\alpha }\sqrt{2\theta_{2}h_{0}^{2}}}{\theta_{1}}+\frac{\theta_{2}h_{0}\left(h_{0}-1\right)}{\theta_{1}^{2}}\right]}^{\frac{1}{h_{0}}}$$

In Equation (10), $d_{\alpha }$ is the deviate belonging to the upper 1−α percentile of the standard normal distribution, while $\theta_{i}$ and $h_{0}$ are metrics derived from the polynomial sums of the p−a eigenvalues of the covariance matrix of the data.

In most industrial applications, the system to be supervised is dynamic, meaning that the observed process data will be of a time-series nature. This, however, results in the presence of significant autocorrelation between subsequent samples, which cannot be accurately described by traditional PCA. The issue of autocorrelation has mainly been addressed through the introduction of various DPCA algorithms, which augment the base method with steps to account for dynamic changes in the data [28].

The most simple approach is the augmentation of the initial data matrix with lagged versions of the process variables to indirectly include autocorrelation in the PCA procedure [29]. This version of the method became generally known as the original DPCA technique and is most widely applied.

### 2.2. Failure Mode and Effect Analysis (FMEA)

The traditional static FMEA method is a “bottom–up” inductive logic-based procedure used to solve quality and reliability issues in the development stages of processes or later for risk assessment of already existing technologies [30]. Base FMEA is at the core of the method; this technique can be specified further to resolve specific issues related to safety and quality with a focus on process (Process FMEA or PFMEA), product design (Design FMEA or DFMEA), system functionality (Functional FMEA or FFMEA) and software issues [15]. The FMEA technique is based on the hierarchical decomposition of the system and identification of failure modes on the lowest possible indenture level. Subsequently, the effect of failure modes on the higher-ordered subsystems is observed and iterated through them [31]. The FMEA analysis can be enhanced through a subsequent criticality evaluation of identified failure methods. The FMEA analysis should result in the following items:

- Systematic overview of possible failures;
- Evaluation of failure impact of the system performance;
- Identification of failure causes;
- Quantitative evaluation of risks associated with each failure mode;
- Specification of corrective actions for risk reduction.

The FMEA procedure is initiated by consulting relevant professionals who have sufficient empirical and theoretical knowledge of the observed process. The specific topic of FMEA is established (the scope of the observed system), and system specifics such as system architecture, characteristics, and functions are analyzed by the professionals. For a reliability and risk analysis, all possible system failure scenarios are evaluated. The failure modes are most commonly identified based on observed issues of similar systems, based on historical data or in case of novel processes system decomposition and analysis techniques, such as product–function analysis, function–component relationship analysis, function–structure relationship analysis, etc., during brainstorming sessions of the team of professionals. More rigorous methods such as fault tree (FTA) or event tree analysis (ETA) are also often times applied to perform the failure mode analysis [32].

After analyzing the failure modes and their propagation through the system, the risk evaluation of each failure method is compiled. The risk evaluation of failure modes in FMEA can be performed in a wide manner of ways, with the most common being the application of the risk priority number (*RPN*) [31]. This involves either the addition or multiplication of three factors associated with the failure mode, these being severity (*S*), occurrence (*O*), and detectability (*D*). In order to express these scores, two main approaches are utilized, these being the expression of risk factors S,O,D through fuzzy logic, the other being the use of a 10-level integer scale to quantify each measure [30]. The *RPN* score using the latter solution is calculated traditionally as per Equation (11):

(11) RPN=S·O·D

While the FMEA method is a great tool to ensure process quality and safety in the design stages of a system, it lacks capabilities for online diagnosis, therefore limiting its capabilities for system supervision and decision-making [33]. Therefore, FMEA is often enhanced or fused using more rigorous risk assessment techniques with a probabilistic framework to enable system diagnosis as well. A common approach is the integration of FMEA into Bayesian networks or Markov models [34].

### 2.3. Bayesian Networks (BNs)

BNs are graphical models which are used for representing cause-and-effect relationships using Bayes’ theorem of conditional probability [35].

**Figure 1:**
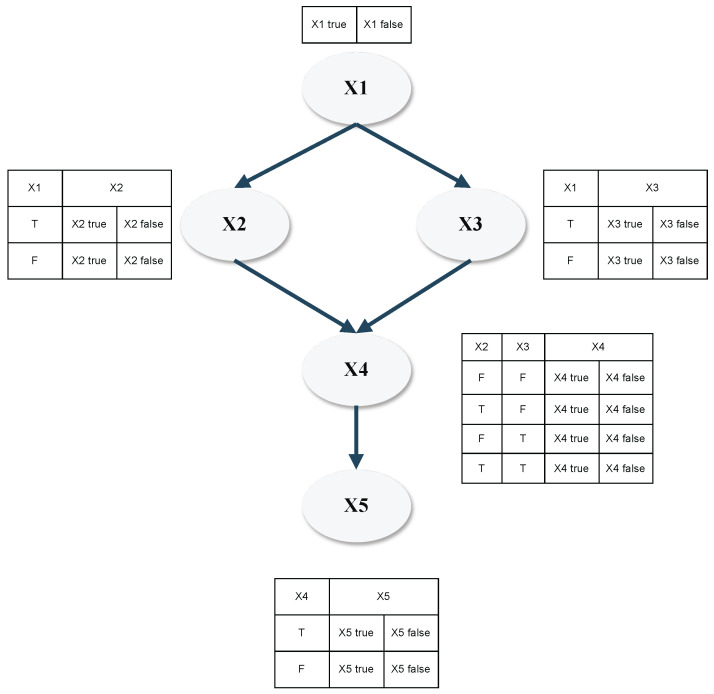
Example of a BN structure with 5 nodes that have binary states.

BNs can be visually represented as directed acyclic graphs (DAGs), where root nodes are root causes of a series of events linked by causality, and leaf nodes are the final possible consequences of the root event (an example is provided in Figure 1).

The nodes of BN are linked through conditional probability functions (CPFs); in general, the CPF can be a continuous or discrete probability distribution function, the former describing an infinite number of possibility values for process states for a node variable, and the latter assuming only a certain amount of fixed probability values for the variable states. In the risk estimation state, the possible event states are usually discrete variables (for example, “Is the *i*-th system component faulty? “ → the corresponding states can be “true” or “false”); here, conditional probabilities determine each states’ likeliness based on known event states within the DAG [36]. The individual CPFs of different states are summarized in conditional probability tables (CPTs) describing the conditional dependence of all states of a node, given all possible states of its parent nodes. To provide an example, in Figure 1, the CPTs are displayed next to each node, depicting the conditional probabilities of node states based on their parent nodes; binary states “True-T” and “False-F” are given for each node.

During the quantitative analysis of the net, the user wishes to estimate the probability of a given state of a variable in the BN through knowledge about the state of another observable variable by means of inference. For this, the conditional dependence of the variables in the network has to be analyzed. We denote the CPF of a given variable *x* on another variable *y* as CPF(x|y). For any given variable in the graph, the probability distribution of its states may be calculated using the probability distribution of its parent nodes’ states through the underlying assumptions of conditional independence encoded in the graph using Equation (12):

(12) $$CPF\left(x_{1},x_{2},\cdots ,x_{i}\right)=\prod_{j=1}^{i}CPF\left(x_{j}\;|\;pa\left(x_{j}\right)\right)$$

The structure of BNs can be approximated using expert knowledge or data-based techniques, the same being true for the various CPFs and CPTs. In our work, the structure of the BN is based on the initial FMEA analysis of the system, while the CPFs are approximated using maximum likelihood estimation [37] (where simulation data are available) and expert knowledge in cases which are not observed during the simulation.

## 3. The Proposed Technique for Prediction of Safety-Critical Events

**Figure 2:**
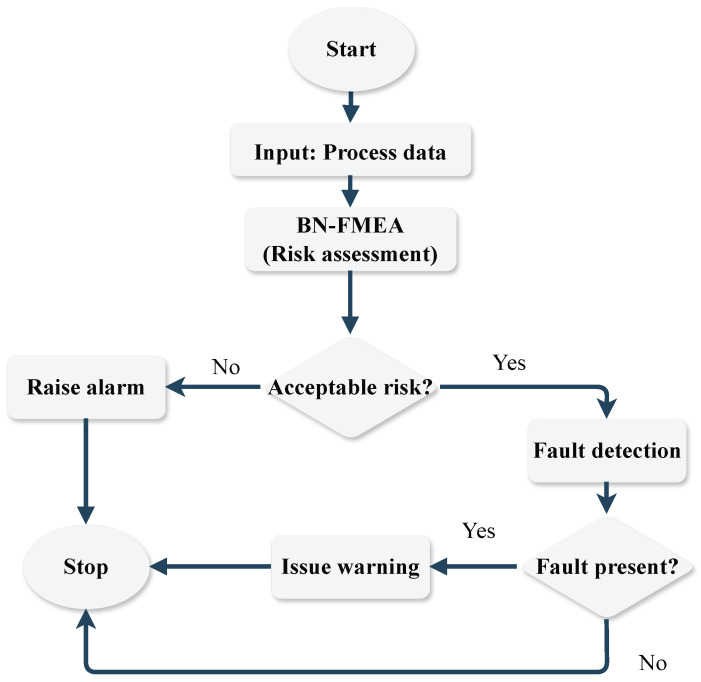
Flowchart of the proposed risk-based FD algorithm.

The outline of the proposed online supervision strategy is shown in Figure 2.

To set up the procedure, first, the structure of the system is evaluated through FMEA analysis. Possible failure modes are summarized, and operation data are acquired through the analysis, the resulting FMEA chart serving as a baseline for the BN structure. Generally, the concept of inferring a BN from FMEA is not a novel idea; many approaches and tools have been proposed, yet the use of expert knowledge is the most basic solution to establish the subsequent BN [38].

In this work, the FMEA results are translated into BN, and the CPFs are established through expert knowledge and maximum likelihood estimation using historical data where applicable. This means that possible failure modes as identified by FMEA serve as root nodes for the network, associated failure symptoms are derived from said root nodes and are linked to the observable variables of the process. The causal relationships between the nodes are established using simulation data. Conditional probability functions between root causes and the resulting symptoms are identified using maximum likelihood estimation based on generated simulation data. The DPCA model is established through simulation results; accounted faults are the failure modes identified through the FMEA procedure. As such, for each failure mode, missed alarm rates are calculated. Missed alarm rates serve as the basis for the detectability scores of the *RPN* calculations.

During the online supervision process, the real-time system behavior is observed. The occurrence probability of failure modes could be directly calculated from the observed process variables as symptoms of the function of time. The detectability of individual failure modes based on the missed alarm rate and severity scores assigned based on expert knowledge are used to create an *RPN* score for each possible failure mode. Should the risk level for any failure mode indicated by the *RPN* exceed the acceptable range, alarms are instantly raised, while in the case of no serious process risk, the evaluation of the fault presence is performed. Thus, for real-time risk assessment, the detectability derived from DPCA, the occurrence probability of failures estimated from the observed process variables, the Bayesian network, and the severity assigned using expert knowledge and FMEA analysis aer combined. If an observed anomaly holds no significant process risks, it is still analyzed, and warnings are issued if faults are detected based on the DPCA results, but alarms are not raised. If no faults are present, no immediate actions are taken.

## 4. Case Study and Method Evaluation

The method is tested using data gathered from a three-tank benchmark system and for a model of a dehydrogenation reactor utilized in LOHC technology. The results are displayed in the following subsections.

In case of the three-tank benchmark, the entire procedure starting from DPCA model development until the BN and FMEA establishment is thoroughly explained. In the LOHC case study, the procedure is showcased, but the thorough procedure is not explained in detail, as it is identical to the steps described in the three-tank benchmark problem.

### 4.1. Case Study of the Three-Tank Benchmark Problem

**Figure 3:**
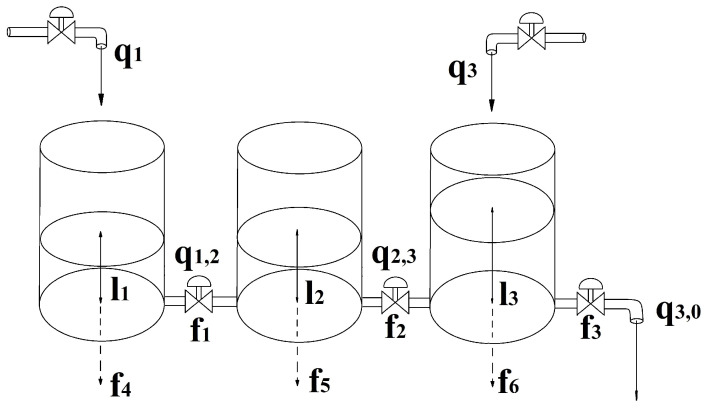
Scheme of the investigated three-tank system [39].

The performance for risk-based FD of the proposed method is evaluated using data obtained from the predefined model of the three-tank benchmark system shown in Figure 3 [39]. This benchmark problem is widely recognized and frequently cited in FD literature for testing various methods [40,41]. A comprehensive description of the benchmark problem, including the measured input and output variables, system model, and system parameters, is provided below.

#### 4.1.1. First Principle Model of the Three-Tank Benchmark

The investigation focuses on a system comprising three linked cylindrical tanks in succession, each sharing a consistent cross-sectional area denoted as *S*. Positioned at the base of each tank is an outlet. Connecting these tanks are cylindrical pipes, all possessing a cross-sectional area designated as $S_{n}$, enabling the flow of fluid between them.

The system’s measurable output variables encompass the liquid levels within each tank, denoted as $\left(l_{1},l_{2},l_{3}\right)$. The influencing factors on these levels are the inlet flow rates into the first and last tanks, represented as $\left(q_{1},q_{3}\right)$. Flow rates between tanks are symbolized as $q_{i,j}$, where the indices *i* and *j* represent the connected tanks or the external environment (0). These rates are contingent on the liquid levels within the tanks and the outflow coefficients $\mu_{i,j}$, regulated by the valve positions in the pipe segments.

To validate the methodology, six potential faults are scrutinized. The initial three faults involve a reduction in the outflow coefficients within each pipe segment $\left(f_{1},f_{2},f_{3}\right)$, possibly stemming from sediment accumulation or control valve dysfunctions. The subsequent three faults pertain to leakages in each tank $\left(f_{4},f_{5},f_{6}\right)$.

A fundamental model of the system is constructed to gather data on its performance under various normal and faulty conditions. The computation of flow rates between tanks follows Torricelli’s law. The system of differential equations describing liquid levels as well as the calculation of the flow rates can be seen in previous articles detailing the benchmark problem [39,42].

To ascertain the volumetric flow exiting the tank due to leaks $\left(q_{i,f}\right)$, fault signals $f_{i+3}$ are binary, with a value of one indicating a leakage and zero indicating no leakage. The model undergoes simulation to collect data under normal conditions. A steady-state for the system is designated, and adjustments to the input variables (inlet flow rates) are executed to monitor changes in the tank water levels. Subsequently, these data are utilized in developing the DPCA transformation.

The operational parameters of the chosen steady state, alongside the tank construction parameters and potential fault parameters, are detailed in Table 1.

**Figure 4:**
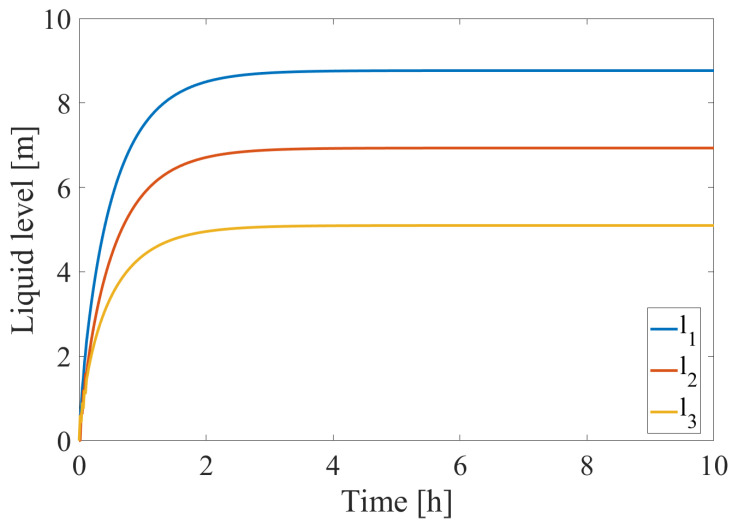
Steady-state liquid level within the three tanks under the operating conditions of Table 1.

The training data for the DPCA method are obtained by observing system behavior around the steady state under the conditions shown in Table 1. The development of the steady-state conditions can be seen in Figure 4. The system of differential equations describing liquid level changes is solved using Euler’s explicit method.

#### 4.1.2. Dynamic Principal Component Analysis for Fault Detection

The system was observed in a time window of 1200 h, with a sampling time of 600 s. The observation period and sampling time was chosen based on the time-constant of the system which was approximately 5 h. During this time, 30 changes were made in the value of the input volumetric flow according to a ramp function. The deviation of the inputs from their steady-state values were calculated as random variables with a normal distribution according to Equation (13), where *m* is the expected value of set point change (0 in this case), σ is the standard deviation of change (0.01 in this case), and *N* is a random variable following the standard normal distribution:

(13) $$u=u_{steadystate}\cdot \left(m+\sigma N\right)$$

**Figure 5:**
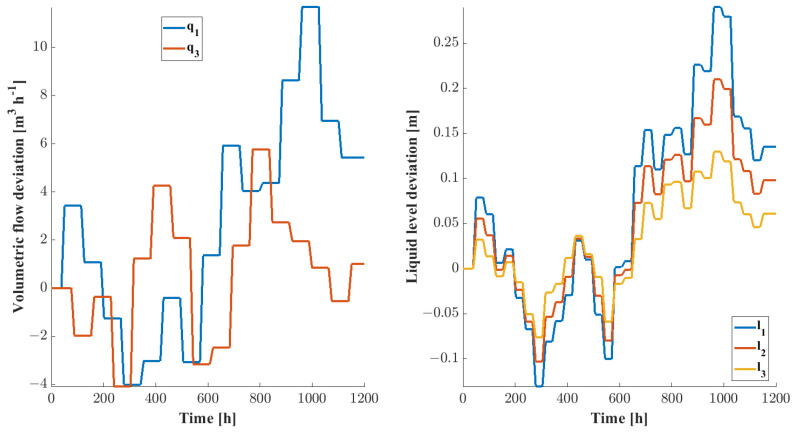
Changes in the inlet volumetric flows (**left**) and the liquid levels (**right**) within the tanks compared to their steady-state conditions.

The changes in the input volumetric flow and the liquid levels compared to their steady-state values are shown in Figure 5.

The DPCA model was constructed in accordance with the algorithm proposed by Ku et al. in the original article detailing DPCA [24]. The PCA transform was calculated as per Equation (3) to Equation (6). The lag number was tuned as well as the number of PCs. To determine linear relations, a threshold was determined for the eigenvalues ($\lambda_{min}$) of the corresponding PC scores. The chosen threshold for the eigenvalues was determined using Equation (14):

(14) $$\lambda_{min}=\max_{1\leq i\leq p}\lambda_{i}\cdot 10^{-4}$$

**Figure 6:**
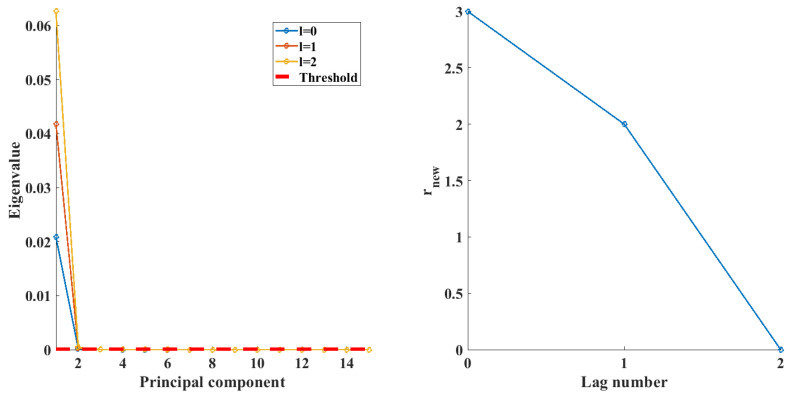
Eigenvalues of the PCs (**left**) and the number of new relations for different lag values (**right**).

During the tuning, lag values from 0 to 2 are utilized, for testing. The eigenvalues of the DPCA transformation as well as the threshold are plotted for the investigated instances, the results of which can be seen in Figure 6, in the form of a scree plot. As the lag number increases, the numeric values of the first few eigenvalues also increase; therefore, at lag 2, the third PC also becomes more significant. Based on the results, an optimal lag number of 2 is determined, and the first two PCs are retained. The $r_{new}$ value of new relations is calculated during the different iterations and plotted, the results of which can be seen in the second subplot of Figure 6. The subplot shows that the transform reveals new successive relations due to the addition of lags, and the autocorrelation between data is properly contained within the PCs; however, after a lag number of 2, no new relationships can be observed.

**Figure 7:**
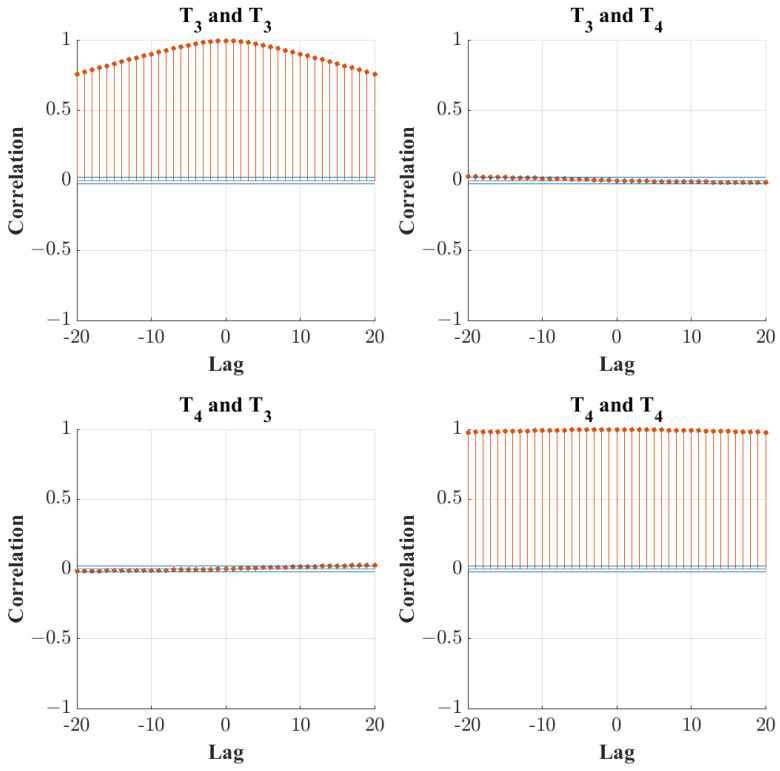
Autocorrelation plots for the first three discarded PCs in basic PCA.

**Figure 8:**
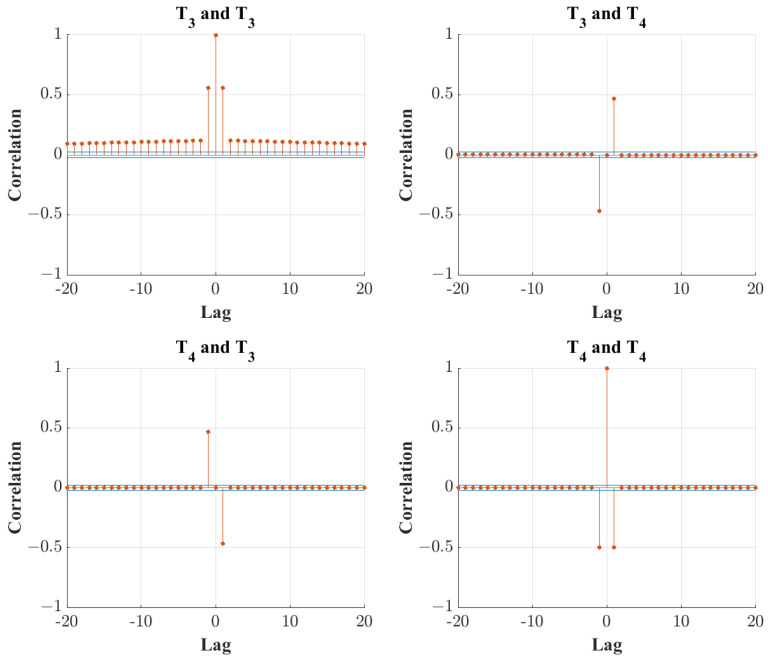
Autocorrelation plots for the first three discarded PCs in basic DPCA, with a lag number of 2.

The auto and cross-correlation in the discarded PCs is observed to validate the results as proposed by Ku et al. [24]. The results for the first two discarded PCs for both the original PCA transform and the chosen DPCA transform with a lag value of 2 are displayed in Figure 7 and Figure 8. It must be noted that for 0 lags, the optimal PC number to be retained is 1, and thus, the correlation plots are displayed for PCs 2 to 3. When comparing Figure 7 and Figure 8, it is shown that the DPCA transform with 2 lags significantly decreased the autocorrelation of the discarded PC scores, meaning that the dynamic tendencies of the data are mostly captured in the transform.

**Figure 9:**
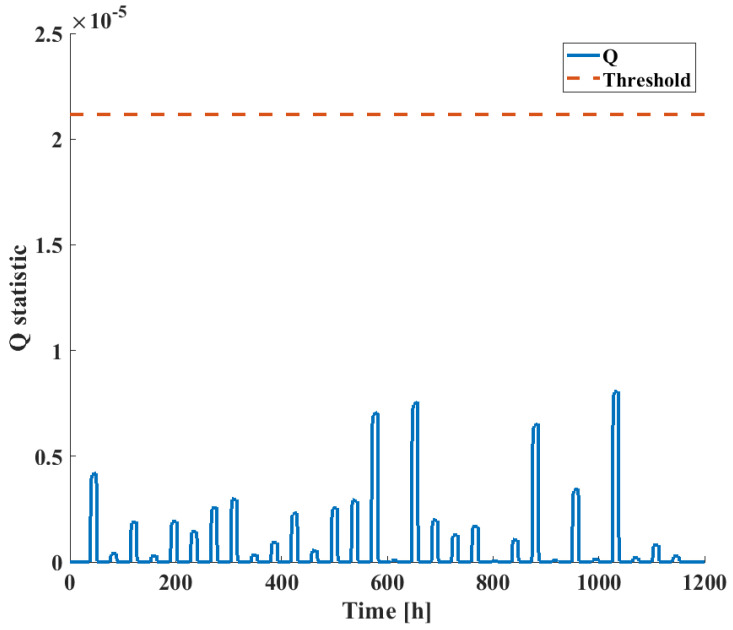
*Q*-statistic for original training data (Figure 5) with the trained DPCA model.

To validate the performance of the model on the training data set, the *Q* statistic was calculated and evaluated against the upper control limit calculated from Equation (10), corresponding to a 95 % confidence level. The results are shown in Figure 9; the low value of the *Q* statistic and the fact that it nowhere exceeds the control limit indicates that the model accurately represents the process.

#### 4.1.3. Risk Assessment of the Three-Tank Benchmark System

The FMEA analysis for the unit was performed with a focus on process functionality; the core was a PFMEA evaluation. Using the observed system in Figure 3, the six main failures used in the case studies were defined as root causes for the observable failure modes. The results of the initial PFMEA analysis for each system component are shown in Table 2. During the analysis, the following assumptions were made:

- The liquid stored in the tanks was water.
- The system contained both liquid level and flow rate measurement sensors.
- Failures of system components were accounted for, but no sensor failure was taken into account.

The possible failures in this case are the degradation of the valve flow coefficient due to fouling and leakages due to corrosion. Both issues can lead to abnormal level changes within the tank. While valve fouling can decrease the flow rate between tanks, leading to overflow, leakages result in direct material outflow from the tank. In both cases, the end effect is water spill, which can lead to human injury through various accidents. The evaluation of severity, observability, and occurrence scores were discretized onto the traditional 10-scale FMEA, using the conditions and criteria displayed in [43].

**Figure 10:**
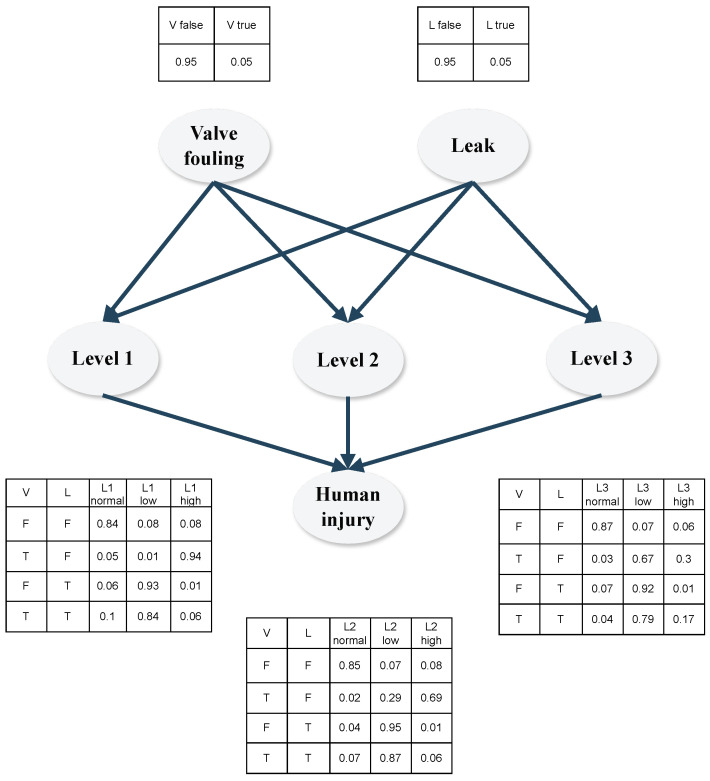
BN for risk assessment of the three-tank system.

Based on the analysis, a preliminary BN was established to model the process. The graphical representation of the BN is shown in Figure 10. The model was developed using the results of the FMEA analysis as well as expert knowledge and process data. The connections between the observed variables (in this case the liquid levels in the tanks) and the fault scenarios were established using historical data through process simulation of 7200 h, with a data set containing 100 setpoint changes and 500 fault scenarios, including simultaneously occurring fault instances. Since each valve and leakage failure mode has the same associated severity and detectability scores, the individual valve and leakage failures ($f_{1}-f_{6}$) were not represented. Using the historical data obtained through the simulations, the CPTs of the liquid level values associated with valve fouling and leakage scenarios were calculated using the maximum likelihood estimation algorithm (MLE) [44]. The probability of valve fouling, leakage and the conditional probability of human injury could not be estimated, as no historical data were available to the authors; therefore, the authors utilized expert knowledge to give estimates for the probabilities.

The valve fouling and leak instances both have two possible states “False-F” and “True-T”, while the liquid level states can be “Low-L”, “Normal-N” or “High-H”. The state of the liquid level scores was assigned using Equation (15), where $m(l_{i})$ and $\sigma (l_{i})$ are the mean and standard deviation of the respective *i*-th liquid level over the simulation interval, and $l_{i}\left(t\right)$ is the *i*-th liquid level at a given time stamp *t*:

(15) $$State_{l_{i}}\left(t\right)=\left\{\begin{array}{cc} L & \;l_{i}\left(t\right)<m\left(l_{i}\right)-\sigma \left(l_{i}\right)\\ N & \mathrm{if}\;m\left(l_{i}\right)-\sigma \left(l_{i}\right)\leq l_{i}\left(t\right)\leq m\left(l_{i}\right)+\sigma \left(l_{i}\right)\\ H & \mathrm{otherwise} \end{array}\right.$$

Subsequently, the risk profile of each failure mode could be continuously calculated as a function of time through Equation (11). Since the severity and observability of both faults modes is known a priori and statically, the time-dependent part of the *RPN* score is the probability of a fault mode occurring. Using the BN, the risk of valve fouling and leakage are constantly calculated using the PCs. Thus, for each failure mode, an *RPN* risk profile can be observed, and an acceptable *RPN* threshold can be given. In the following, the results of the method for the three-tank system are shown through a case study with a timescale of 300 h and 5 simulated fault scenarios.

**Figure 11:**
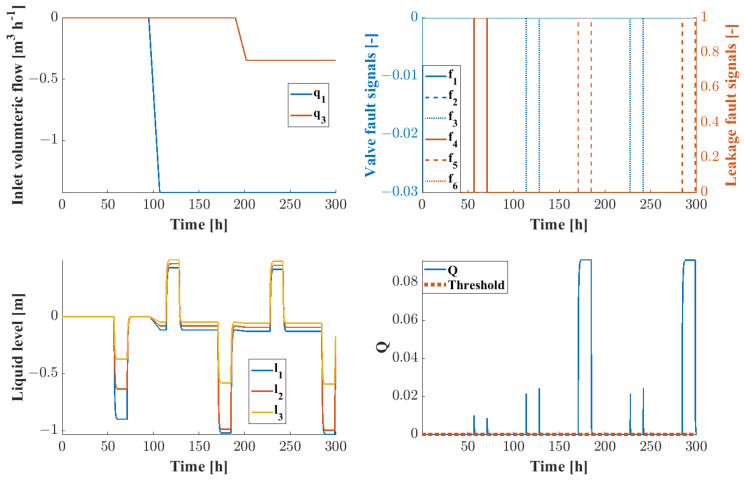
Changes in inlet volumetric flow (**upper left**), system fault signals (**upper right**), system level changes (**lower left**) and values of the *Q* statistic for the three-tank benchmark problem (**lower right**).

**Figure 12:**
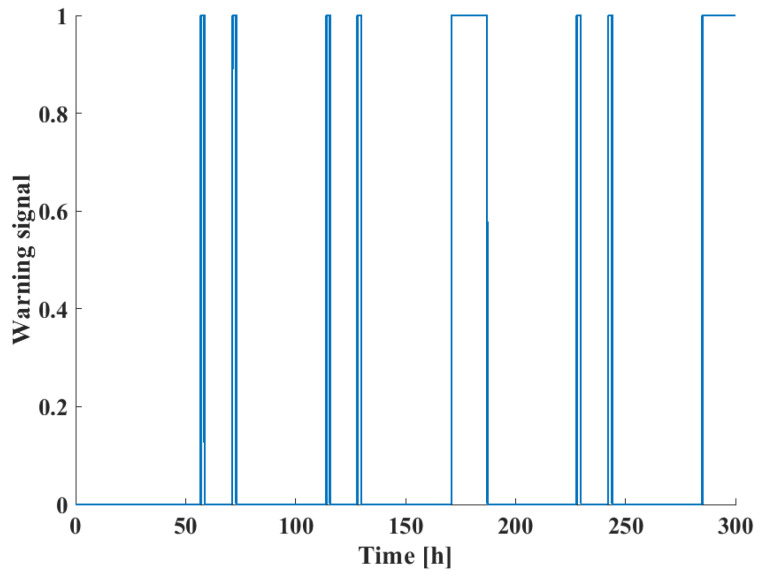
Warning signals for the case study of the three-tank benchmark problem.

Figure 11 shows the changes in the input volumetric flow of the system as well as the values of the fault signals over the observation period. Also displayed are the changes in liquid level, compared to their steady-state values, and the values of the Q statistic for FD. It can be seen that the greater values of the Q statistic correspond well to the fault signals. The warning signals of FD, seen in Figure 12, when the Q statistic exceeds the statistic limit correspond to the fault signals. After running a simulation of 10,000 h time with 1000 randomly generated fault signals and 50 set point changes, the FAR and MAR values were estimated to be 1.7 and 12.9%, respectively.

**Figure 13:**
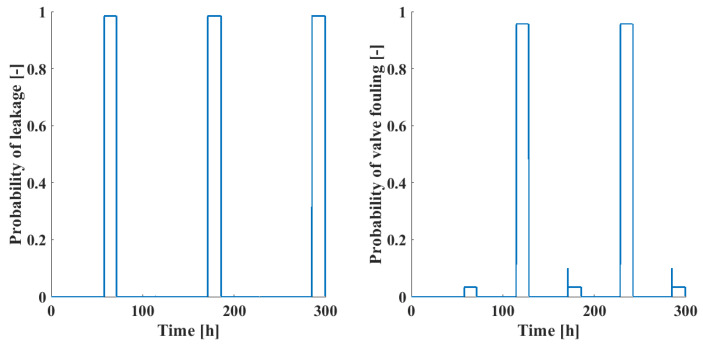
Probability of leakage (**left**) and valve fouling (**right**) failure modes in the three-tank benchmark problem.

Using the values of the liquid levels, the probability of failure modes was calculated using the BN structure of Figure 10. The resulting probabilities are shown as a function of time in Figure 13. When comparing Figure 13 with the fault signals in Figure 11, it can be seen that both leakages and valve fouling can be reliable identified using the BN; in the case of leakages, the distinction is almost perfect, while in the case of valve fouling, instances of leakages also result in small probability values of valve fouling but the actual valve fouling events possess significantly higher probability scores.

**Figure 14:**
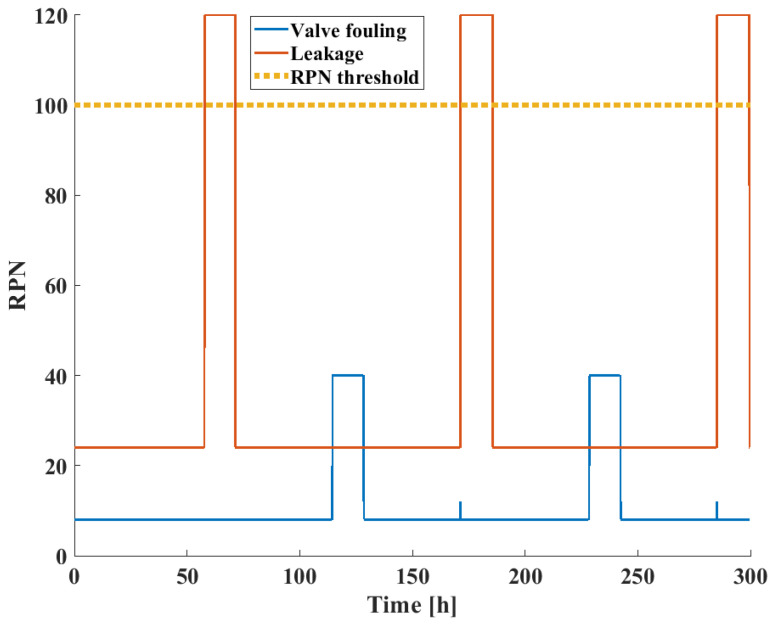
*RPN* scores of failure modes in the three-tank benchmark case study.

The *RPN* scores as a function of time are shown in Figure 14 for both failure modes. In the case of valve fouling, the low probability events which belong to leakages induce no great differences in the final *RPN* score, while actual valve fouling events are characterized by the maximal possible *RPN* for this failure mode (40). In the case of leakages, the accurately identified leakage events all achieve their maximum *RPN* value (120). The *RPN* threshold for this application is also shown; it was chosen as 100.

**Figure 15:**
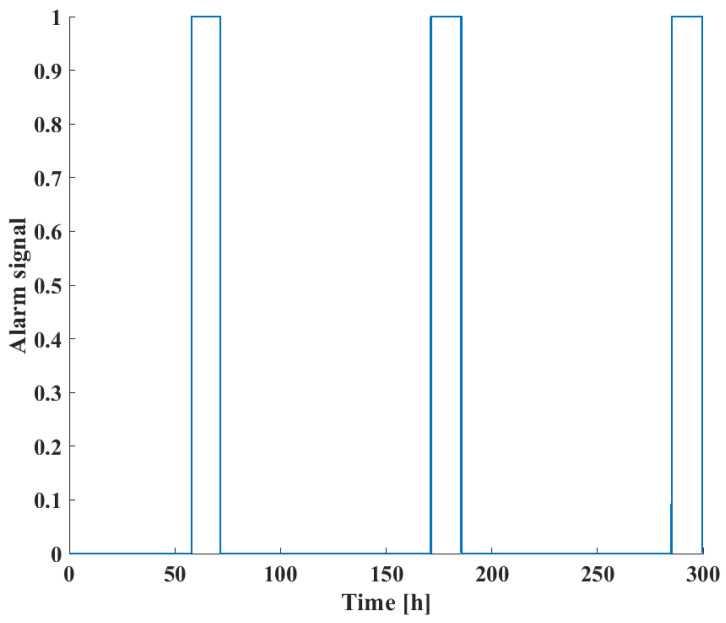
Alarm signals in the three-tank benchmark case study taking both process risk and FD results into account.

Finally, the actual alarm signals are shown in Figure 15, taking both process risk and FD results into account. When compared with the warning signals in Figure 12, it can be seen that while all fault instances may be reliably detected using the DPCA technique, the FMEA-based BN risk analysis was able to sort out safety-critical events, which require the attention of operators and possible shutdowns of the system to prevent accidents in the technology.

### 4.2. Case Study of the Liquid Organic Hydrogen Carrier (LOHC) Technology’s Dehydrogenation Reactor

The LOHC technology is a promising industrial process for the safe storage and transportation of hydrogen, which is fundamental for the hydrogen-based economy [45]. One of the critical questions of hydrogen-based energy involves the safe and economically sustainable transport and storage of hydrogen during its lifecycle. The transport and storage of hydrogen as a low density and highly explosive gas is a critical issue [46]. Various solutions have been proposed for this problem, such as binding hydrogen to metal-hydrides or storing hydrogen as a high pressure gas or in a liquified state. As an alternative to these techniques, the LOHC process for hydrogen transport and storage involves chemically binding hydrogen during a reaction to a liquid organic carrier molecule for safe transportation, which can be economically beneficial, as it allows storing hydrogen at ambient conditions [45]. The transport of hydrogen during this procedure is based on two steps: the first involves the binding of the hydrogen (hydrogenation) into the LOHC molecule, and the subsequent, second step is the release of hydrogen (dehydrogenation) at the site of use.

#### 4.2.1. First-Principle System Model of the Dehydrogenation Reactor

In this case study, we have studied the dehydrogenation step of an LOHC reactor with methyl cyclohexane ($C_{7}H_{14}$, from now on MCH) as a carrier molecule. During the dehydrogenation step, the transported hydrogen ($H_{2}$) is removed from the carrier in a heterogeneous catalytic reaction, leading to the formation of toluene ($C_{7}H_{8}$, from now on TOL) and $H_{2}$ [47]. The formula of the reaction is shown in Equation (16):

(16) $$C_{7}H_{14}\to C_{7}H_{8}+3H_{2}$$

In this study, the kinetics of the reaction were assumed to follow the Langmuir–Hinshelwood–Hougen–Watson (LHHW) kinetics, which is suitable for heterogeneous reactions in the presence of a solid catalyst [48]. Parameters of the kinetic equation were identified using experimental data from a laboratory-scale plug flow dehydrogenation reactor.

**Figure 16:**
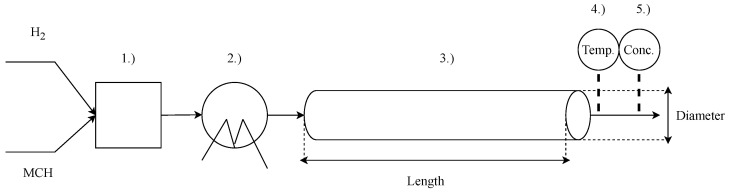
Simplified layout of the reactor system.

In our case study, we utilized a simplified structure of the system. The assumed layout of the studied unit is displayed in Figure 16. The reactor is fed $H_{2}$ and MCH, and the gas streams are mixed before entering the system in the mixer unit (1.), in which their concentration ratio is controlled. After creating the proper mixture, the gas stream is heated in a heat exchanger (2.) to the temperature of the operating point before entering the reactor (3.). The reactor is an adiabatic plug flow reactor where the dehydrogenation process takes place. After exiting the reactor, the temperature (4.) and concentration of $H_{2}$ and MCH in the outlet stream are measured using a sensor unit (5.).

The constructional parameters of the pilot reactor such as length (*l*), diameter (*d*), cross-section area (*A*) and volume (*V*) are shown in Table 3.

Observed variables within the reactor are the concentration of $MCH,H_{2}$ within the feed as well as the inlet temperature $T_{in}$, and the concentrations of the components at the outlet as well as the outlet temperature $T_{out}$.

Using the identified kinetic parameters, a first-principle model for the system was developed. The flow regime was approximated as being an ideal plug flow. During the calculations of energy and component mass balance, the convection (in the longitudinal direction) and source terms due to reaction were accounted for. Under the above assumptions, the component mass and energy balances for the unit were given as a system of partial differential equations shown in Equation (17):

(17) $$\begin{array}{c} \frac{\partial c_{i}}{\partial t}=-v_{x}\frac{\partial c_{i}}{\partial x}+r_{i}\\ \frac{\partial T}{\partial t}=-v_{x}\frac{\partial T}{\partial x}+\frac{\sum_{i=1}^{N}\Delta H_{r,i}r_{i}}{\rho c_{p}} \end{array}$$

In the equation, $c_{i}$ refers to the concentration of the *i*-th component, $v_{x}$ is the flow velocity in the longitudinal direction within the reactor, $r_{i}$ is the reaction source term for a specific component, $\Delta H_{r,i}$ refers to the reaction heat of specific reactions taking place, and ρ and $c_{p}$ are the density and heat capacity of the medium within the reactor.

The mathematical model of the system was solved using MATLAB R2020b, with the appropriate initial and boundary conditions. The initial (x,t=0) and boundary (x=0,t) conditions as well as the parameters of the material within the unit are seen in Table 4, where *B* is the inlet volumetric flow rate of the feed. The dependence of heat capacity and density of the material was studied as a function of temperature, and it was found that in the investigated regime, the material qualities showed no significant changes. In light of this, both the density and heat capacity of the material system were assumed to be constant during the investigations.

Should the flow conditions in the system not allow the use of ideal system models such as plug flow, then alternatively, computational fluid dynamics methods may be utilized to obtain data pertaining to system behavior. In the case of material properties such as density or heat capacity, when these greatly vary over the observation period, then experimental functions may be fitted to account for their changes due to temperature fluctuations. While these changes may cause increased computational loads for data generation, they have no impact on the procedure of the proposed supervision algorithms. If DPCA performance were to deteriorate, then alternative non-linear methods such as kernel principal component analysis (KPCA) may be used to characterize system behavior, estimate missed alarm rate, and perform fault detection.

#### 4.2.2. Risk Assessment for the Dehydrogenation Reactor

In the following, the application of the proposed method for the LOHC case study is discussed. An FMEA analysis was initiated to pinpoint safety-critical failure modes of the system. During the analysis, the following assumptions were made:

- The system contains outlet temperature and concentration sensors. Faults in the sensors were not taken into account during the FMEA analysis.
- A heat exchanger is present at the inlet of the unit, which heats the inlet mixture to the desired temperature. No heat exchanger is present, however, along the length of the reactor.

In the light of these assumptions, the FMEA table is shown in Table 5.

The characteristic safety indicators of the process are the changes within the mixture temperature and the concentration of hydrogen and MCH. Thus, the risk level of the process is determined based on the deviation of these three variables. The root causes for the deviations include the failure of the process heat exchanger due to fouling, which causes deviation of the process temperature from its nominal values; this, in turn, can lead to catalyst deactivation within the reactor unit.

**Figure 17:**
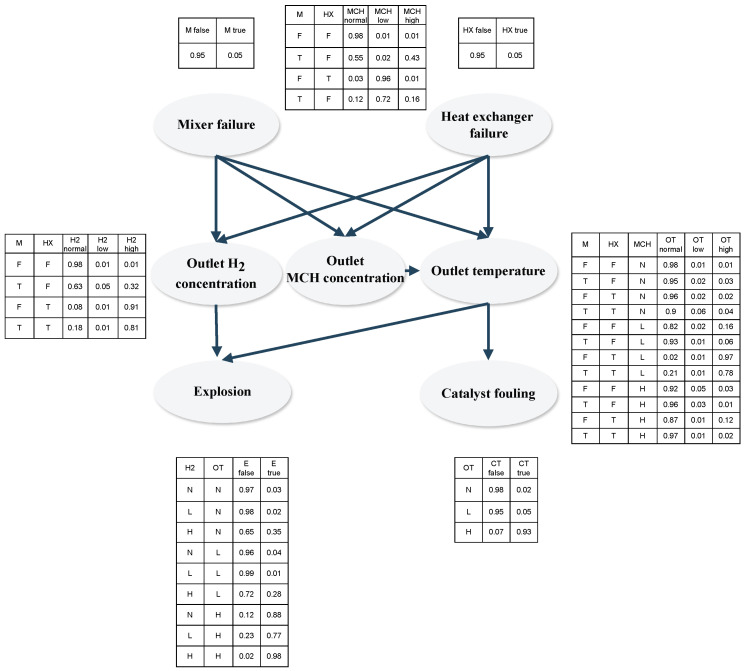
BN for risk assessment of the LOHC dehydrogenation reactor.

In the case of abnormal MCH or hydrogen concentrations being simultaneously present due to mixer control failure, this could lead to possible explosions. The BN established through the FMEA analysis is shown in Figure 17.

Mixer failure and heat exchanger failure as well as explosion and catalyst fouling have two possible states “False-F” and “True-T”, respectively; the CPTs of these occurrences, similarly to the previous instance, were filled out using expert knowledge, as no process data were initially available. In contrast to this, the relationship between the failure modes and the failure symptoms (inlet concentration and outlet temperature deviation) were filled out using simulation case studies as before through the use of maximum likelihood estimation. The failure symptoms have three possible states “Normal-N”,“Low-L” and “High-H” respectively which were determined similarly as Equation (15).

After training the DPCA model using observation data of the process obtained for 1000 h using a set of 500 observed process faults and 100 set point changes, the FMEA-based BN was utilized to simultaneously detect faults and observe process risks. There are three distinct types of process faults, $f_{1MCH}$ being the fault of the mixer causing changes within the MCH inlet concentration, $f_{1H_{2}}$ being the change in the inlet $H_{2}$ concentration due to mixer failure, and $f_{2}$ being the fault of the heat exchanger resulting in abnormal outlet temperature.

**Figure 18:**
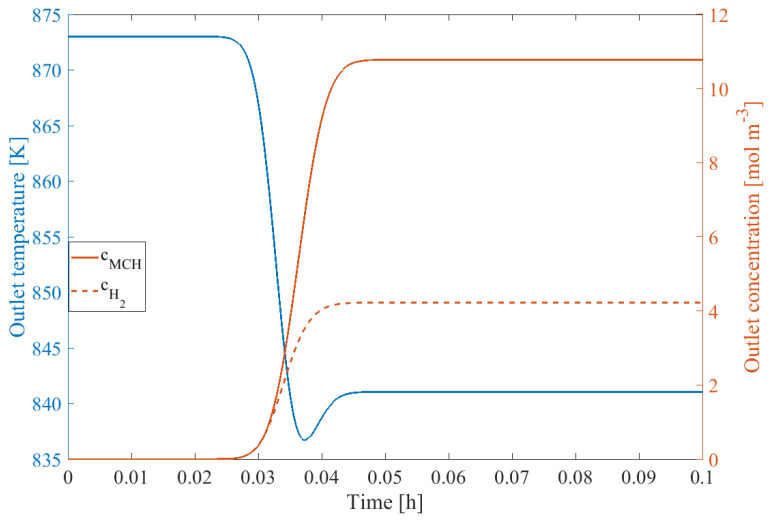
Steady-state operating point of the LOHC technology under the conditions in Table 4.

The steady-state concentration and temperature profile of the unit are shown in Figure 18 under the conditions given in Table 4.

**Figure 19:**
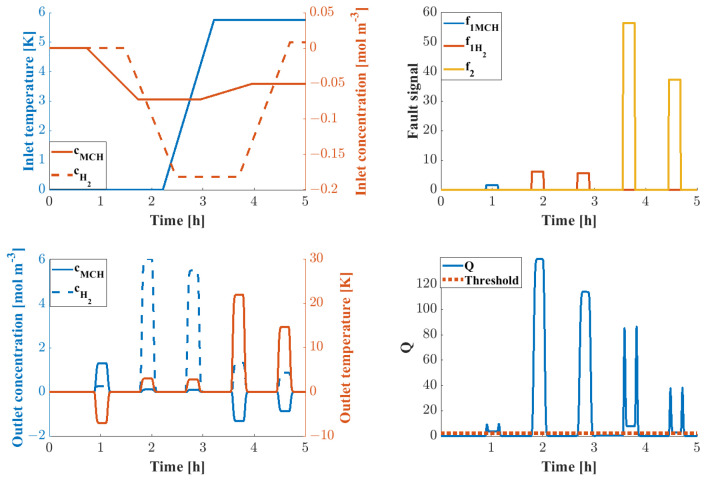
Fault detection procedure of the LOHC process, operating point changes (**upper left**), fault signals (**upper right**), system responses (**lower left**), and *Q* statistic (**lower right**).

The changes within the steady-state boundary conditions and the possible fault signals are shown in Figure 19 under an investigation of 1 h of simulation time with 5 set point changes and 5 fault signals. It can be seen that all faults could be isolated using the *Q* statistic within reason.

**Figure 20:**
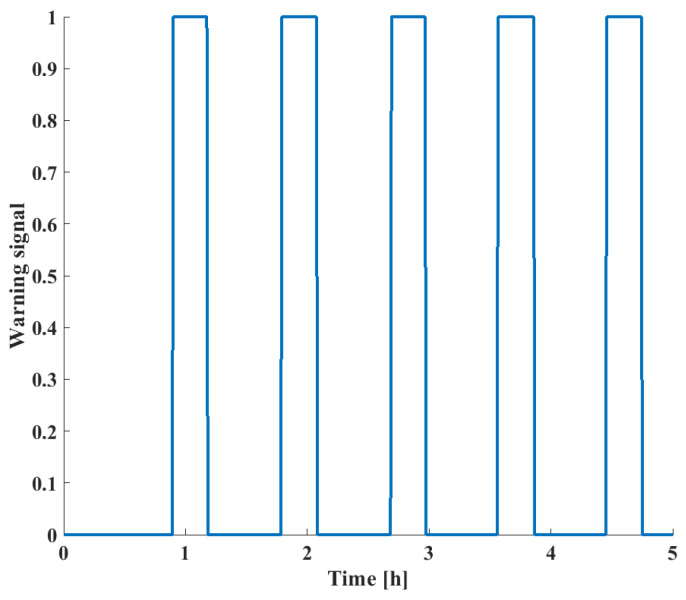
Warning signals given based on the DPCA FD procedure.

The warning signals due to fault presence are shown in Figure 20; the warning signals correspond to the fault presence.

**Figure 21:**
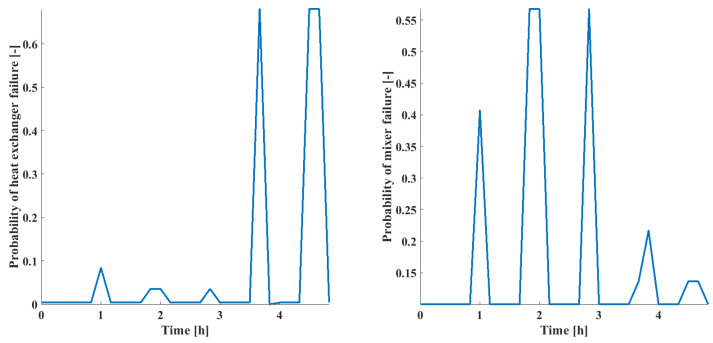
Probability of heat exchanger (**left**) and mixer (**right**) failure modes as a function of time.

The risk of each failure mode was calculated using the trained BN; the results for both the heat exchanger and mixer failure are displayed. The probability of each failure mode as a function of time is displayed in Figure 21.

**Figure 22:**
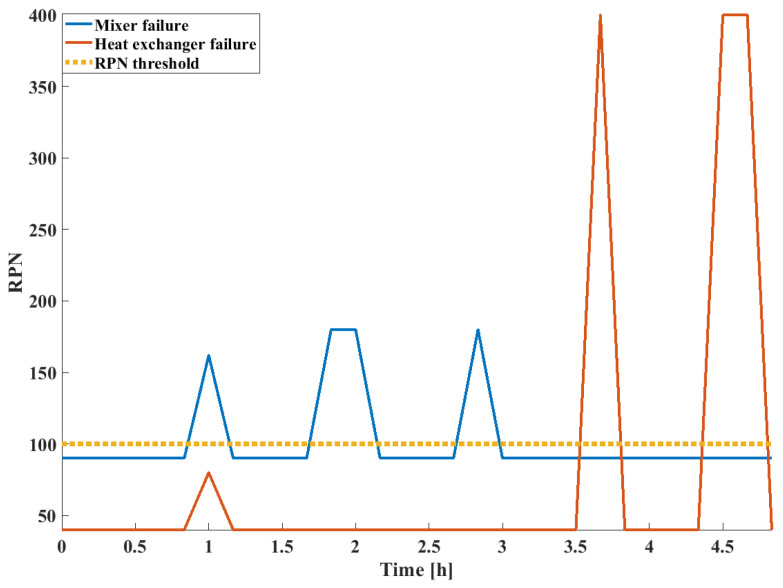
*RPN* score as a function of time.

The corresponding *RPN* scores are shown in Figure 22.

**Figure 23:**
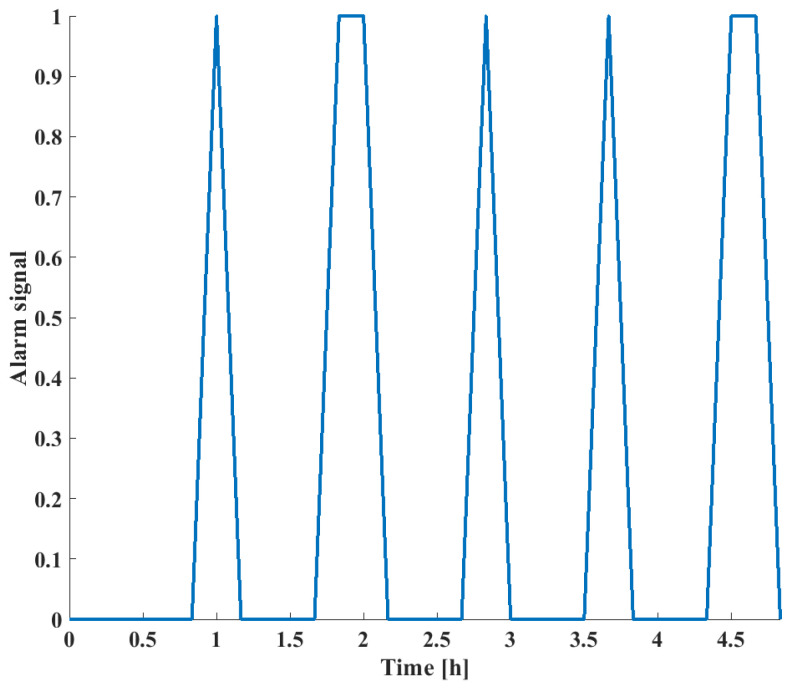
Alarm signals as a function of time for the LOHC reactor.

Finally, the alarm signals based on the *RPN* score are shown in Figure 23.

Comparatively, it can be seen that the change in *RPN* scores of different failure modes correspond to the actual failure scenarios shown in Figure 19. The *RPN* scores are defined by the extent of deviation of the given process variables from their expected steady-state values; in this case, all three process faults carried significant risks. However, as seen in Figure 15, when non-safety-critical faults are present, they are eliminated by the *RPN* screening. This way, the FD capabilities of the system are not decreased since warnings will indicate fault presence; however, alarm floods can be prevented, as only critical failures are highlighted.

This was tested in the case of both studies. In both instances, 10,000 fault random fault signals were generated, and the alarm and warning numbers were compared. In the case of the three-tank system, the ratio of alarms to warnings was 0.63, with a MAR of 12.5%. For the LOHC example, the alarm-to-warning ratio was 0.89 with a MAR of 4.3%. In both instances, the number of alarm signals was significantly decreased; only safety-critical faults were highlighted, while faults which posed no significant risks were effectively filtered out as warnings.

## 5. Discussion

Conclusively, the results show that the method could effectively diagnose systems, pinpoint the presence of faults, and differentiate between safety-critical and non-safety-critical process abnormalities.

Compared with previously introduced methods, the technique has the advantage of being based on standard risk assessment techniques (FMEA), which is widely available for industrial applications. In addition, opposed to previous works, where risk was calculated based on a probability of system malfunction and associated severity scores, in this study, the modified *RPN* for risk assessment took fault propagation paths, fault detectability, and severity into account, integrating the established frameworks of FMEA, Bayesian networks, and DPCA.

Through the tunable *RPN* threshold, the safety restrictions can be relaxed or increased, effectively alternating between recognizing all faults as warnings or alarms (the latter case being the use of DPCA results, as they are for alarm raising).

## 6. Conclusions

In this work, the authors introduced a risk-based fault detection (FD) method which utilizes dynamic principal component analysis for FD and a Bayesian network (BN), constructed using failure mode and effect analysis (FMEA) as a risk assessment tool.

The method was used for the online supervision of systems and was showcased using a three-tank benchmark model and the model of a laboratory scale reactor used during the dehydrogenation step of the liquid organic hydrogen carrier (LOHC) technology. In both cases, the method managed to effectively reduce the number of process alarms by filtering out non-safety-critical process faults, thus reducing the possibility of alarm floods. The reduction in superfluous alarm signals was between 11 and 37%, respectively, for the investigated case studies.

On average, the use of the technique reduced the number of raised alarms by 20–30% in the observed case studies while being sensitive enough to pinpoint all fault presences.

## Figures and Tables

**Table 1:** Operational and constructional parameters of the investigated system.

$q_{1}\;\left[m^{3}s^{-1}\right]$	$q_{3}\;\left[m^{3}s^{-1}\right]$	$\mu_{1,2}\;\left[-\right]$	$\mu_{2,3}\;\left[-\right]$	$\mu_{3,0}\;\left[-\right]$
$1.5\times 10^{-4}$	$1.5\times 10^{-4}$	0.5	0.5	0.6
$\mu_{f}\;\left[-\right]$	$S\;\left[m^{2}\right]$	$S_{p}\;\left[m^{2}\right]$	$S_{f}\;\left[m^{2}\right]$	$l_{i,0}\;\left[m\right]$
0.6	$1.5\times 10^{-2}$	$5\times 10^{-5}$	$5\times 10^{-5}$	0

**Table 2:** FMEA table of the three-tank benchmark system.

Fault Root Cause	Function	Potential Failure Mode	Potential Cause of Failure	Failure Consequences	Current Process Controls	Recommended Actions	Severity Score (S)	Detectability Score (D)
Valve	Flow rate control	Decreased flow rate	Valve fouling	Overflow, Human injury	Flow rate sensor	Valve Cleaning	2	2
Tank	Liquid containment	Leakage	Corrosion	Human injury	Liquid level sensor	Tank welding	3	4

**Table 3:** Geometrical parameters of the reactor.

Construction Parameter	Value
*l* [m]	4.5 $\times \;10^{-1}$
*d* [m]	1.4 $\times \;10^{-2}$
*A* [$m^{2}$]	2.3 $\times \;10^{-4}$
*V* [$m^{3}$]	1.02 $\times \;10^{-4}$

**Table 4:** Initial and boundary conditions as well as material parameters within the unit.

Boundary Conditions	Values	Initial Conditions and Material Parameters	Values
*B* $\left[m^{3}\;s^{-1}\right]$	5.5 $\times \;10^{-6}$	$c_{MCH}$ $\left[mol\;m^{-3}\right]$	0
*v* $\left[m\;s^{-1}\right]$	3.5 $\times \;10^{-3}$	$c_{TOL}$ $\left[mol\;m^{-3}\right]$	0
$c_{MCH}$ $\left[mol\;m^{-3}\right]$	12	$c_{H_{2}}$ $\left[mol\;m^{-3}\right]$	0
$c_{TOL}$ $\left[mol\;m^{-3}\right]$	0	*T* [K]	593
$c_{H_{2}}$ $\left[mol\;m^{-3}\right]$	3	ρ $\left[kg\;m^{-3}\right]$	2.99
*T* [K]	593	$c_{p}$ $\left[J\;kg^{-1}K^{-1}\right]$	0.23

**Table 5:** FMEA table of the LOHC benchmark system.

Fault Root Cause	Function	Potential Failure Mode	Potential Cause of Failure	Failure Consequences	Current Process Controls	Recommended Actions	Severity Score (S)	Detectability Score (D)
Heat exchanger	Temperature control	Abnormal temperature profile	Heat exchanger fouling	Explosion, catalyst fouling	Outlet temperature sensor	Heat exchanger cleaning, process shutdown	9	2
Mixer	Inlet concentration control	Abnormal inlet concentration profile	Valve sticking	Explosion, product loss	Inlet composition sensor	Shutdown, valve change	10	4

## Data Availability

No new data were created or analyzed in this study. Data sharing is not applicable to this article.
